# Biventricular Mechanical Circulatory Support Does Not Prevent Delayed Myocardial Ventricular Rupture following Myocardial Infarction

**DOI:** 10.1155/2013/767541

**Published:** 2013-03-18

**Authors:** Yazhini Ravi, Emily P. Sudhakar, Pratima Nayak, Chittoor B. Sai-Sudhakar, Konstantinos Dean Boudoulas

**Affiliations:** ^1^Division of Cardiac Surgery, Department of Surgery, The Ohio State University, 420 W 12th Avenue, Columbus, OH 43210, USA; ^2^Division of Cardiovascular Medicine, Department of Medicine, The Ohio State University, Columbus, OH 43210, USA; ^3^College of Medicine, The Ohio State University, Columbus, OH 43210, USA

## Abstract

Cardiogenic shock and myocardial rupture can complicate an acute myocardial infarction (AMI). A case is reported in which a 58-year-old male with an acute inferior myocardial infarction required placement of biventricular assist device for hemodynamic support eight days after the onset of his AMI; eleven days after his AMI, the patient developed abrupt onset of hemodynamic instability with massive bleeding from his chest tube due to delayed free wall myocardial rupture that was discovered when he was taking emergently to the operating room. Myocardial rupture in patients with a ventricular assist device should be considered in the differential diagnosis in the event of acute hemodynamic compromise. A high level of suspicion for such a complication should prompt aggressive and emergent actions including surgery. We present a case of delayed free wall myocardial rupture following an acute inferior wall myocardial infarction in a patient with biventricular mechanical circulatory support.

## 1. Introduction

Ventricular free wall rupture is a fatal complication after myocardial infarction. With an increase in the management of cardiogenic shock with mechanical circulatory support devices, there should be a high level of suspicion for mechanical complications after myocardial infarction. The possibility that the VAD may contribute to the rupture also needs to be explored.

## 2. A Case Report

A 58-year-old male with a history of coronary artery disease presented to an outside hospital with an acute inferior wall myocardial infarction and cardiogenic shock. An emergent cardiac catheterization was performed and a bare metal stent was placed to the right coronary artery. An intra-aortic balloon pump (IABP) was inserted due to hemodynamic instability. Further, an Impella 2.5 (ABIOMED, Inc., Danvers, MA) cardiac assist device was also placed via a percutaneous approach from the femoral artery. A transthoracic echocardiogram performed demonstrated severely dilated ventricles with severe biventricular systolic dysfunction. The patient at that time was transferred to our facility, a large tertiary medical center, for further management. Due to continued cardiac decompensation upon arrival, the IABP and Impella 2.5 were removed, and the patient was placed on venoarterial extracorporeal membrane oxygenation (ECMO) using the femoral vessels.

**Figure 1:**
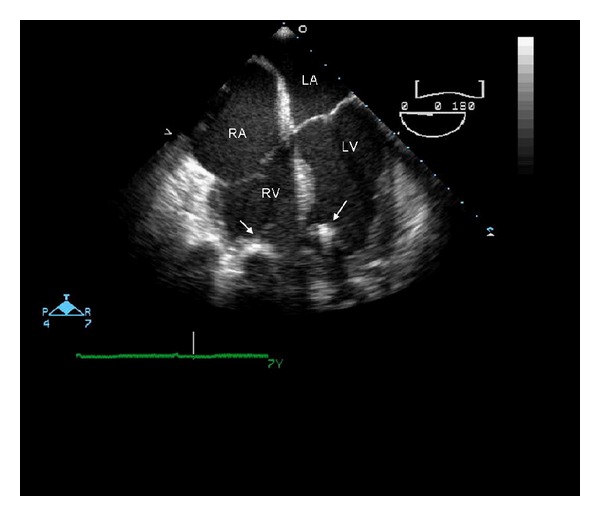
Transoesophageal echocardiogram 4 chamber view: biventricular assist device inlet cannulas in the left and right ventricles are shown (arrow). RA: right atrium, LA: left atrium, RV: right ventricle and LV: left ventricle.

The patient continued to require hemodynamic support with ECMO eight days after his myocardial infarction; thus, a decision was made to provide biventricular mechanical support as a bridge to heart transplantation. In the operating room, the ECMO circuit was explanted and biventricular assist devices (BIVADs) (Figure 1) were placed using the Thoratec Implantable Ventricular Assist Device (Thoratec Corporation, Pleasanton, CA). Cannulation of the right ventricle and pulmonary artery were used for the right ventricular assist device, while the left ventricular apex and the ascending aorta were used for the left ventricular assist device (Figure 1). The patient was hemodynamically stable using the BIVAD support.

Eleven days following his initial presentation and three days after the BIVAD placement, the patient developed acute hypotension with massive (approximately 2 liters) bleeding from his chest tubes. The patient was emergently taken to the operating room with a provisional diagnosis of cannula dislodgement. In the operating room, the patient was placed on emergent cardiopulmonary bypass. On entering the pericardial cavity, copious bleeding was observed from the diaphragmatic surface of the heart. Aggressive suctioning of the blood demonstrated a 5 cm × 5 cm necrotic area with myocardial rupture on the inferior myocardial wall of the left ventricle corresponding to the distribution of the right coronary artery and away from the cannulas. The myocardial rupture was repaired successfully using Teflon strips and large caliber sutures. The patient was weaned-off cardiopulmonary bypass, and the BIVAD support was reinstituted. Unfortunately, a computed tomography scan of the brain that was obtained after surgery demonstrated diffuse cerebral edema with global ischemia. After discussing with family members, supportive comfort care measures were instituted, and the patient expired three days after his operation for his myocardial rupture repair.

## 3. Discussion

The complications of acute myocardial infarction may include cardiac arrhythmias, ventricular dysfunction with varying degrees of heart failure, mechanical complications including papillary muscle rupture leading to acute mitral regurgitation, free wall myocardial rupture, and ventricular sepal defect in the zone of infarct [1]. Myocardial rupture results from a transmural infarction and is associated with hemodynamic collapse resulting in a high mortality [2, 3]. The incidence of myocardial rupture is rare and is responsible for approximately 7% of in-hospital deaths due to a myocardial infarction. Myocardial rupture typically occurs within 3 to 5 days of infarction during the prethrombolytic era; however, with the use of thrombolytic therapy, rupture usually occurs between 24 and 48 hours of infarction [1]. The incidence of myocardial rupture decreases in patients with acute myocardial infarction treated with primary percutaneous coronary intervention, and usually if occurs, it is seen within the first day after infarction [4]. Factors contributing to myocardial rupture include ST-segment elevation myocardial infarction, advanced age, female gender, and hypertension [6]. Signs of myocardial rupture include tachycardia and hypotension [5]. Early detection and prompt surgical intervention can prevent death due to a myocardial rupture complicating acute myocardial infarction.

Cardiogenic shock after an acute myocardial infarction secondary to severe left or right ventricular systolic dysfunction is associated with a mortality of approximately 59% and 55%, respectively [7]. With increasing evidence demonstrating poor survival in this patient population without mechanical circulatory support devices, consideration is given to the early institution of circulatory support by percutaneous or open surgical techniques. Newer devices have the ability to provide cardiac outputs ranging from 1 to 10 liters per minute and decrease the workload of the ventricle allowing for recovery of the left ventricle or provide valuable time and stabilization of the patient to explore other options including long-term support or heart transplantation. Our patient developed cardiogenic shock following a transmural infarction of the inferior wall of the left ventricle and required biventricular mechanical support. While the literature reports indicate that the clinical presentation of myocardial rupture is observed early during the course of an acute myocardial infarction, the delayed presentation (11 days after myocardial infarction) in our patient was considered to be unusual. With the increasing use of mechanical circulatory support in the management of acute decompensated heart failure, mechanical complications of acute myocardial infarction should be carefully considered in the differential diagnosis, particularly when alterations in the flow characteristics of the mechanical assist device occur or if there is a sudden decompensation of a patient on full mechanical circulatory support. We hypothesize that the presence of BIVADs may have delayed the onset of this complication as the decompression of the left ventricle reduces the wall stress and the propensity of the necrotic area to rupture. The other possibility which was considered was that Thoratec BIVAD is unique in that it incorporates an active intermittent suction mechanism to empty the ventricle that may perhaps result in repeated stress on the area of infarction contributing to rupture.

Myocardial rupture complicated by an acute myocardial infarction may be delayed in patients with ventricular assist devices. Myocardial rupture in patients with a ventricular assist device should be considered in the differential diagnosis in the event of acute hemodynamic compromise. A high level of suspicion for such a complication should prompt aggressive and emergent actions including surgery.
